# Disentangling the pseudoknots of toxin translation

**DOI:** 10.1073/pnas.2411591121

**Published:** 2024-07-18

**Authors:** Jonathan Jagodnik, Fabien Darfeuille, Maude Guillier

**Affiliations:** ^a^Microbial Gene Expression department, UMR8261 CNRS, Université Paris Cité, Institut de Biologie Physico-Chimique, Paris 75005, France; ^b^University of Bordeaux, Department of Technology for Health, INSERM U1212, CNRS UMR 5320, ARN: Régulation Naturelle et Artificielle (ARNA) Laboratory, Bordeaux F-33000, France

Toxin–antitoxin systems (TA) are genetic modules encoding both a toxic protein, at least when overexpressed, and its antitoxin antidote. Such systems were originally discovered on plasmids where initial studies showed the role of these killer genes in plasmid maintenance (1). With the advent of genome sequencing, many homologous systems were then discovered on almost every bacterial chromosome (2). Since then, the identification of new TA systems has grown steadily. Up to 8 types of TA have been classified based on the nature and mode of action of the antitoxin (3). In the case of the type 1 TA (T1TA), the antitoxin is an RNA molecule that inhibits the expression of the toxin gene via pairing to its messenger RNA (mRNA). Several T1TAs were identified serendipitously during the search for small noncoding RNA in bacteria (4). Because the antitoxin inhibits the synthesis of the toxin rather than its activity in T1TAs, it is crucial to keep the toxin expression tightly controlled. A remarkable feature of T1TAs is that, in contrast to most bacterial genes, transcription and translation of the toxin gene are uncoupled. This uncoupling prevents translation of the toxin and is achieved through cotranscriptional folding to mask the Shine-Dalgarno sequence (SD) in the primary transcript within stable intramolecular structures (5, 6). Therefore, an activating step is required to switch the toxin mRNA into a translationally competent form, which is then targeted by the antitoxin, most often via pairing to the liberated SD sequence. In other cases, the antitoxin targets an upstream ribosome binding site, also termed standby site (7), or the SD of an upstream open reading frame, to which translation of the toxin gene is coupled (8).

**Fig. 1.**
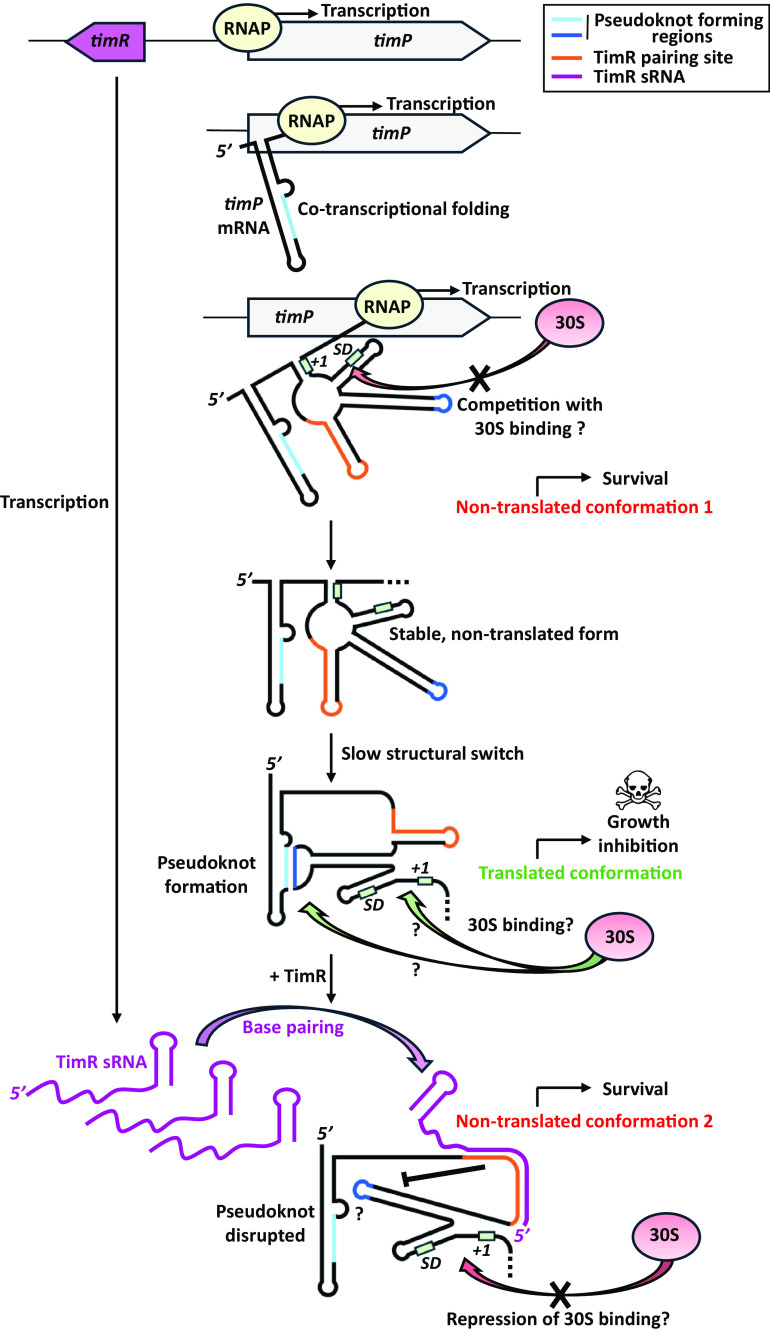
Model of multistep mechanism of *timP* translation initiation regulation proposed by Eleftheraki and Holmqvist (11). *timP* mRNA folds cotranscriptionally, adopting a structure that prevents transcription–translation coupling. This averts TimP protein synthesis and toxicity. Over time, *timP* mRNA 5′UTR structure slowly switches to adopt a pseudoknot conformation that activates translation presumably by promoting 30S binding, leading to TimP toxin synthesis. This translationally active conformation requires a secondary control mechanism conveyed by the TimR sRNA to maintain minimal TimP production. Upon base-pairing to *timP* mRNA, TimR disrupts the pseudoknot, likely preventing 30S binding. mRNA regions forming the translation initiation region (SD and +1 translation initiation position) are depicted in green. RNAP, RNA polymerase.

In Gram-negative bacteria, most toxin mRNAs from T1TAs switch to the translationally active form through a processing event that partly removes the 5′ or 3′ end region of the mRNA. This cleavage leads to some structural remodeling allowing access of the small ribosomal subunit 30S to the ribosome-binding site. However, for several type 1 toxin mRNAs, a processing event could not be detected (9). This suggests that other possible mode(s) of activation to a translation-competent mRNA remain to be characterized (10). In this issue of PNAS, the study of Eleftheraki and Holmqvist has now largely addressed this question for the *timP*-TimR T1TA of *Salmonella* (11). In this system, conserved in enterobacteria, the *timP* toxin gene encodes a small inner membrane protein of 38 amino acids that inhibits growth when overproduced. The divergently transcribed TimR small RNA (sRNA) relieves toxicity by pairing to *timP* mRNA and repressing its translation (Fig. 1) (9). As for other T1TAs, the SD region of the toxin-encoding *timP* mRNA is sequestered, but surprisingly *timP* is translated at detectable levels, both in vivo and in vitro. This confirms that no processing is required for translation activation. Combining structural, biochemical and genetic approaches, Eleftheraki and Holmqvist clearly show an interaction between two regions of the structured *timP* 5′UTR forming a pseudoknot that activates translation.

In addition to this new role for a pseudoknot structure, the authors also analyze *timP* regulation by TimR. Strikingly, an in vitro translation assay revealed a complete repression of *timP* by TimR even though only a minor fraction of *timP* mRNA interacts with TimR, even when this sRNA is in large excess. Binding of TimR to *timP* follows two rate kinetics, with a fast initial binding, followed by a much slower one. Together, these data suggest a model in which TimR preferentially binds the translation-competent pseudoknotted conformation of *timP* mRNA, and this has been experimentally confirmed. In other words, the switch of *timP* mRNA to the pseudoknot conformation is kinetically slow, while the binding of the TimR antitoxin occurs on a much faster scale. Finally, although TimR binding does not overlap with the region involved in pseudoknot formation, this bimolecular complex destabilizes the intramolecular pseudoknot structure, explaining the translation inhibition by this antitoxin (Fig. 1).

These results provide a novel example of the role of RNA dynamics in gene expression and an alternative mode of toxin mRNA activation that does not involve processing. They also raise several questions that will be interesting to address in future studies. First, it is still unclear how formation of the pseudoknot promotes *timP* translation. The experimental data presented by Eleftheraki and Holmqvist rule out the possibility that this switch simply relieves the sequestration of the SD sequence. Instead, the pseudoknot could lead to 30S recruitment or positioning at the translation initiation site. Interestingly, RNA pseudoknot structures have already been associated with translation and translational control in several instances. One well-demonstrated example is found within the *rpsO* mRNA 5′UTR, which clearly sets a requirement for the ribosomal protein S1 for 30S recruitment (12, 13). Importantly, a role for a pseudoknot structure in translation is also true for the *tisB*-IstR T1TA. Specifically, the 5′UTR of processed and translationally competent form of the *tisB* toxin mRNA contains a pseudoknot structure required for translation initiation (14). This pseudoknot participates in the 30S recruitment to the 5′UTR standby site, possibly through recognition by S1 (14). RNA pseudoknots are also predicted near the 5′-ends of other processed and active mRNAs of T1TAs, such as *zorO* or *shoB*, suggesting that the *tisB* findings may be applicable to other examples (14, 15). Even though the data reported here for *timP* differ from these other T1TAs (as *timP* is a non-processed mRNA), a role for the pseudoknot in recruiting S1, alone or in complex with the 30S subunit, is an appealing hypothesis.

A second relevant question is whether, in a riboswitch-like manner, specific signals or regulators other than TimR could trigger or reduce the formation of the pseudoknot in the *timP* leader and thus modulate toxin translation in response to environmental changes. More generally, an understanding of how the synthesis of the TimP toxin and the TimR antitoxin is controlled, both at the transcriptional or posttranscriptional levels, should be a great help in assessing their physiological functions.

Similar to the *timP* mRNA, there are other type 1 toxin mRNAs, such as *fst* or *txpA* in Gram-positive bacteria, for which no cleavage of the primary transcripts has been identified (10, 16). Given the results reported by Eleftheraki and Holmqvist, it is tempting to speculate that a conformational switch to a translationally competent toxin mRNA could also be the mechanism of activation in the *fst*-RNAII and the *txpA*-RatA systems. At least in the case of *timP*-TimR, this mode of activation leads to a basal expression of the toxin, sufficient to be detected. Hence, it may only apply to proteins with a limited toxicity, which is the case for *timP* that only inhibits growth when overexpressed.

Combining structural, biochemical and genetic approaches, Eleftheraki and Holmqvist clearly show an interaction between two regions of the structured *timP* 5′UTR forming a pseudoknot that activates translation.

Finally, the pseudoknot formed in the *timP* mRNA is one of rare examples of such an RNA motif that is required to activate translation initiation. The difficulty in predicting these types of tertiary interactions in mRNAs probably explains why so few motifs have been described. Indeed, their presence in bacterial mRNAs may be more widespread than expected, especially in the case of highly structured toxin mRNAs. Future studies will be necessary to identify them in genome-wide studies. Another exciting challenge will be to solve the three-dimensional structures of these toxin mRNAs, alone or in complex with their antitoxin, to understand how pseudoknot formation favors not only translation but also antitoxin pairing and, conversely, how this pairing destabilizes the RNA pseudoknot.
